# Multi-Material 3D Printing of a Customized Sports Mouth Guard: Proof-of-Concept Clinical Case

**DOI:** 10.3390/ijerph182312762

**Published:** 2021-12-03

**Authors:** Alexey Unkovskiy, Fabian Huettig, Pablo Kraemer-Fernandez, Sebastian Spintzyk

**Affiliations:** 1 Department of Prosthodontics, Geriatric Dentistry and Craniomandibular Disorders, Charité-Universitätsmedizin Berlin, Corporate Member of Freie Universität Berlin and Humboldt—Universität zu Berlin, 14197 Berlin, Germany; 2 Department of Prosthodontics, Peoples’ Friendship University of Russia (RUDN University), 117198 Moscow, Russia; 3 Department of Prosthodontics at the Centre of Dentistry, Oral Medicine, and Maxillofacial Surgery with Dental School, Tuebingen University Hospital, 72076 Tuebingen, Germany; fabian.huettig@med.uni-tuebingen.de (F.H.); pablo.kraemer-fernandez@med.uni-tuebingen.de (P.K.-F.); 4 Section Medical Materials Science and Technology, Tuebingen University Hospital, 72076 Tuebingen, Germany; Sebastian.spintzyk@med.uni-tuebingen.de; 5 ADMiRE Lab—Additive Manufacturing, Intelligent Robotics, Sensors and Engineering, School of Engineering and IT, Carinthia University of Applied Sciences, 9800 Villach, Austria

**Keywords:** sports medicine, dentistry, polyvinylsiloxane printing, bite guard, additive manufacturing, rapid manufacturing, intraoral scanning

## Abstract

A multilayer mouth guard is known to have the best protective performance. However, its manufacturing in a digital workflow may be challenging with regards to virtual design and materialization. The present case demonstrates a pathway to fabricate a multilayer individualized mouth guard in a fully digital workflow, which starts with intraoral scanning. A free-form CAD software was used for the virtual design. Two various CAM techniques were used, including Polyjet 3D printing of rubber-like soft material and silicone printing using Drop-on-Demand technique. For both methods the outer layer was manufactured from more rigid materials to facilitate its protective function; the inner layer was printed from a softer material to aid a better adaptation to mucosa and teeth. Both 3D printed multilayer mouth guards showed a clinically acceptable fit and were met with patient appraisal. Their protective capacities must be evaluated in further clinical studies.

## 1. Introduction

A mouth guard (MG) is a piece of personal protective equipment which is placed inside the oral cavity to reduce traumatic impact on teeth, mucosa, and alveolar bone during sport activities [1,2]. A large variety of MGs is available on the market today, ranging from trade products to customizable ready-made types to individualized custom-made appliance [3]. Customizable ready-made products are made of thermoplastics, fitted within an oral cavity with the use of the boil-and-bite technique and are the most frequently used [4]. However, their fit and protecting performance is questionable. Custom made MGs were reported to have a higher shock absorbance and a better fit within an oral cavity, allowing for superior wearing comfort [5,6].

An average thickness of 4 mm is considered to be optimal for a MG to withstand the expected traumatic impact while not interfering with the wearer’s comfort [7,8,9,10]. The thickness of 4 mm was tested in material combinations and proved to have a better shock absorbance than multilayer designed MGs [3,11,12]. Today, such multilayer custom MGs are manufactured using an analog method. This involves taking a conventional impression, occlusal vertical dimension (OVD) and registration for stone casts fabrication and articulation [13]. Commonly, a MG is fabricated from ethylene vinyl acetate (EVA) with the use of thermoforming with vacuum moulding [14].

Additive manufacturing (AM) is emerging in the medical field. Computer aided design (CAD) and computer aided manufacturing (CAM) has been successfully applied in prosthodontics, saving time and reducing the cost of production [15,16,17]. While CAD of a multi-layered MG may be feasible for a person with a certain level of expertise, its CAM by means of AM can be regarded as challenging. This would imply a simultaneous printing of both, the inner and outer layers, from biocompatible material with at least two shore hardness grades. Manufacturing a single layer MG in a digital workflow has been reported recently [18]. Another clinical case demonstrated multi-material silicone printing for an extraoral utilization [19]. The feasibility of such technical approaches for an intraoral application has, until recently, remained questionable.

The following clinical “proof-of-concept trial” reports the additive manufacturing of a custom multilayer MG in various Shore A hardness grades within a fully digital workflow, using two types of material: silicone and rubber-like polymer.

## 2. Case Presentation

**Figure 1:**
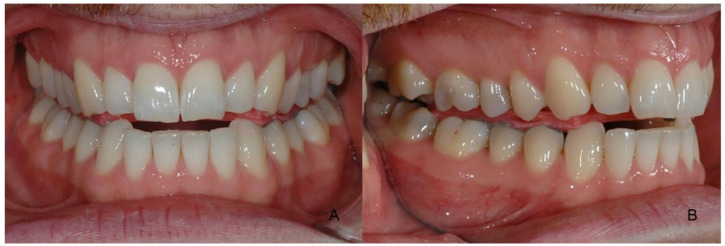
The fixed OVD with a wax plate and Aluwax from the frontal (**A**) and side aspects (**B**).

**Figure 2:**
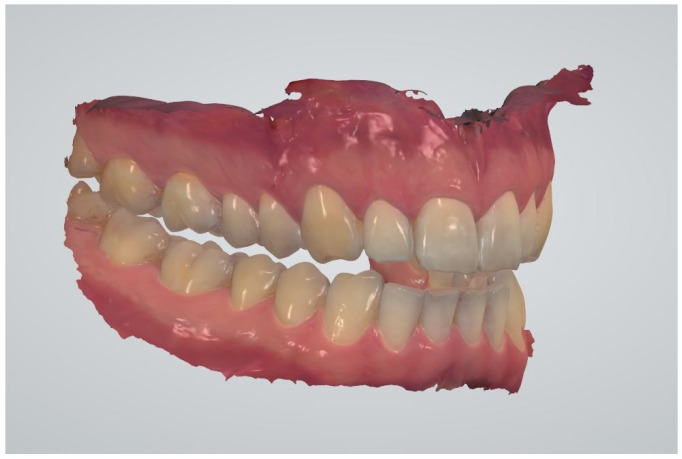
The digital impression of both dental arches with the fixed OVD.

A male patient was referred to the Department of Prosthodontics at the University Hospital Tuebingen in order to be treated with a protective device. After information about potential solutions and current developments, he gave his informed written consent to be provided within a treatment trial with two layered MGs from two types of material to test. The OVD was adjusted in 2 mm vertical raised dimension without pro- or retrusion of the jaw. The position was registered with the wax plate (Beauty Pink Extra Hard 3mm Wax, Integra York PA Inc, York, Pennsylvania, USA) and Aluwax (Aluwax Dental Products Co, Grand Rapids, Michigan, USA) (Figure 1). Thereafter, dental arches were captured digitally with an intraoral scanner (Trios 3, 3Shape, Copenhagen, Denmark) (Figure 2).

The previously determined vertically raised jaw relationship was recorded by two side scans with inserted bite registration. The aligned jaw scans were saved in STL format and uploaded in the free-form CAD software (Zbrush, Pixologic Inc, Los Angeles, California, USA) in order to design a multilayer MG.

**Figure 3:**
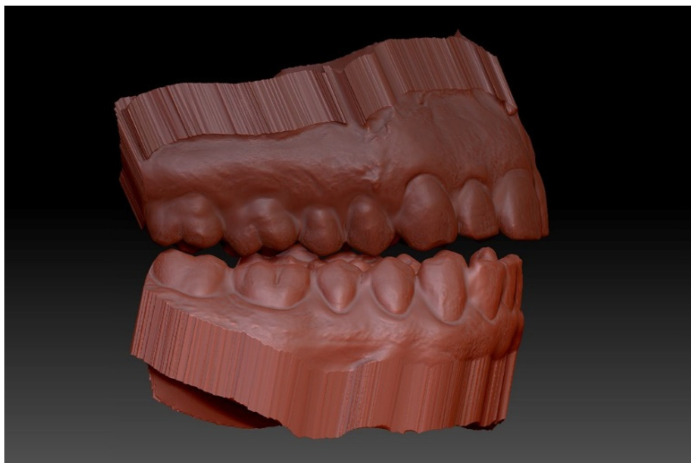
Virtual blocking of anatomical undercuts with the “virtual clay tool” in the Zbrush software.

Firstly, anatomical undercuts were blocked partially with a “virtual clay” tool to a certain extent that it was possible to insert and remove the MG without any problems, while ensuring adequate retention (Figure 3).

**Figure 4:**
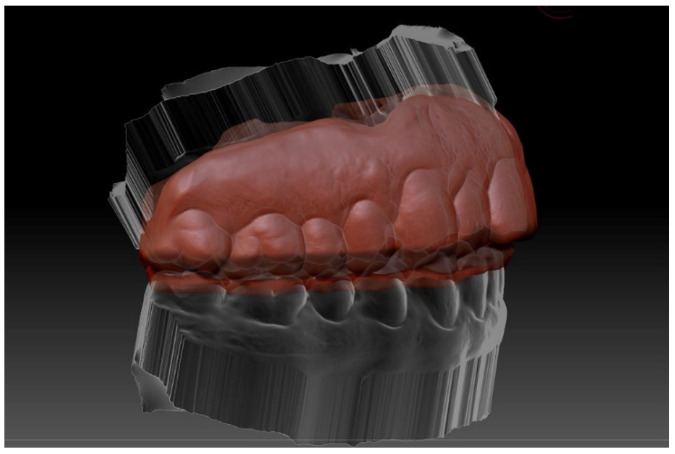
Design of the inner layer (red) (2 mm) using “inflat” tool in the Zbrush software.

For the actual construction of the mouthguard, the “inflat” tool was used to generate two separate layers—inner and outer. To create the “inner layer”, a new object was designed by inflating the surface of the jaw model by 2 mm thickness (Figure 4).

**Figure 5:**
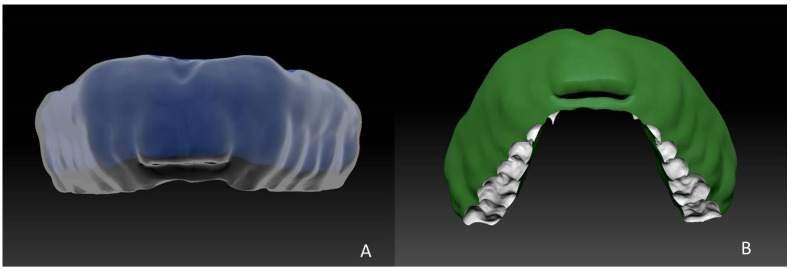
(**A**) Inner layer (blue) and outer layer (transparent) with virtual polishing of the outer surface; (**B**) adaptation of the caudal part to the occlusal surface of the lower jaw and airway creation.

Further on, the “outer layer” was created with the same “inflat” tool by generating a new object and inflating the surface of the original jaw model by 4 mm thickness. To fit the “inner layer” within the “outer layer” the “inner layer” object was subtracted from the “second layer” object with the Boolean-out function, resulting in an “outer layer” of 2 mm thickness. Both layers were checked for any artifacts and voids. The surface of the “outer layer” object, adjacent to oral mucosa, was virtually polished (Figure 5A). The caudal part of MG was adapted to the lower teeth (Figure 5B). A window of 2 × 20 mm in height and width was applied in the frontal part of a MG as an airway for breathing in closed position of the mouth (Figure 5B). Both layers were exported separately in STL format and sent for further CAM.

### 2.1. CAM with Polyjet Polymer

**Figure 6:**
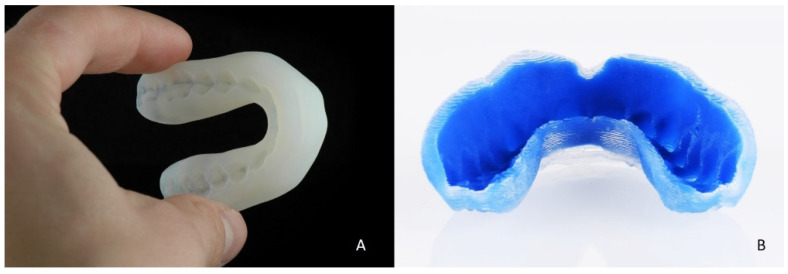
(**A**) The Polyjet printed soft multilayer MG. (**B**) The ACEO silicone printed soft multilayer MG.

The J750 AM machine (Stratasys, Commerce Way Eden Prairie, MN, USA) was used for a multilayer printing of the MG, utilizing the Polyjet technology and a polymer material. The outer layer was printed with rubber-like clear Agilus30 (FLX 4895) as the primary material in 90 Shore A hardness, and the inner layer with clear Agilus30 (FLX 4850) as primary material in 50 Shore A hardness (Figure 6A). In both cases the Rigur was the secondary material. The printing resolution was 16 µm. The printed MG was subjected to post-processing, which implied removing of supporting material.

### 2.2. CAM with Silicone

**Figure 7:**
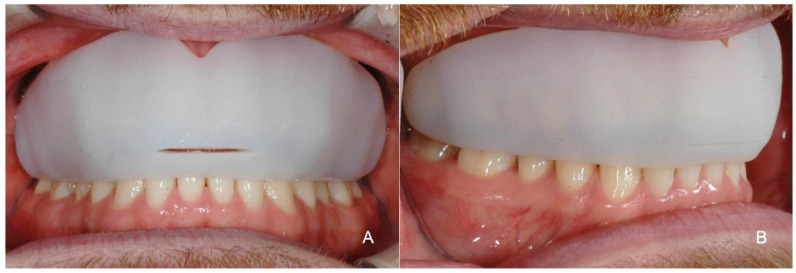
The Polyjet printed multi-material MG in situ, showing a good adaptation to the soft tissue and lower dental arch from the frontal (**A**) and side aspects (**B**). The slit in the middle part shall ensure the air supply, when the teeth are clinched. The outer surface is of micro roughness which may irritate the soft tissues within a short wearing period and calls for further postprocessing.

**Figure 8:**
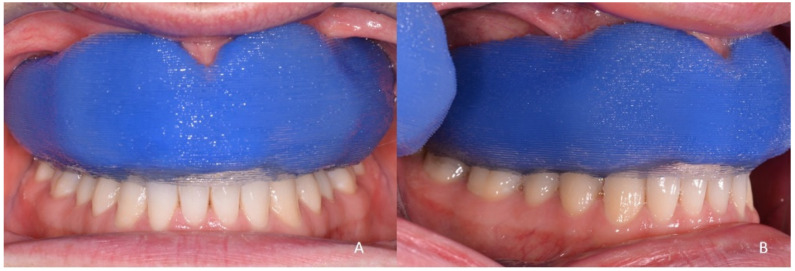
The Drop-on-Demand printed multi-material MG in situ, showing a good adaptation to the soft tissue and lower dental arch from the frontal (**A**) and side aspects (**B**). The staircase effect leaves a macro roughness which is expected to harm the soft tissue over a short wearing period and therefore should be further post-processed.

Alternatively, the same STL data was printed from polyvinylsiloxane using the Drop-on-Demand technique (ACEO), as described previously in the clinical trial by Unkovskiy et al. [19]. The outer layer was printed in 60 A Shore hardness grade and the inner one with 20 A (Figure 6B). Afterwards, both MG were put within the mouth cavity and checked for occlusal relations (Figure 7 and Figure 8). The patient reported comfort wearing both inner and outer surfaces and a reproducible position of the lower jaw, while clenching the teeth

## 3. Discussion

The current trial demonstrates the fabrication of multi-layered MGs in a fully digital workflow using intraoral scanning and multilayer additive manufacturing from two material types. The reported wear comfort may be attributed to the individually adjusted caudal surface of the MG. It can be stated that the digital process chain delivers a visually dimensionally acceptable result within both AM methods. Previous studies have shown that a balanced occlusion and a large number of contacts contribute to the protective effect through minimizing MG displacement [20,21].

With the presented approach, the virtual design of a multilayer MG can be performed within two hours. However, its prerequisites certain CAD skills and financial investments in the freeform software. The state of experimental circumstances can be illustrated by the fact that the applied software version (Zbrush, Pixologic, Version 2020) does not allow direct metric measurements (lineal function) to determine the thickness of a layer for instance. For this reason, a 5 mm 3D cube was uploaded and measured in units. A units-to-millimetre ratio was calculated and applied for further measurements. In consequence, commercial software packages should cover or even automize this (commonly) basic tools to enable a straight-forward-design. The virtual design of the applied MG considered also a window in its frontal part for a better breathing capacity. Whether this additional window indeed contribute to a better breathing and does not compromise the shock absorption of a MG may be tested in further research to this topic.

Additive manufacturing of a biocompatible silicone in 20A, 40A and 60A Shore hardness has been reported recently [19]. However, higher Shore A values starting from 85A are required for a sufficient shock absorbance [12].

Regarding the polymer-based MG, the inner layer adjacent to the teeth and gums was printed in 50 Shore A hardness to ensure comfort for the wearer. The outer surface was executed in 90 Shore A hardness in order to ensure the protective function.

In case of polyvinylsiloxane (ACEO), the inner layer was printed in 20A Shore hardness grade and was met with better appraisal by the patient in terms of wearing comfort compared to 50A of the polyjet polymer material. However, as mentioned above, currently the Drop-on-Demand technology enables the silicone printing of up to 60A Shore hardness, which might not be rigid enough for the outer layer in terms of its protective function. Alternatives may arise with AM-based free-form moulding of medical silicones.

The Agilus30 is a rubber-like material, which is not allowed for permanent contact with soft tissue. In contrast, while the ACEO polyvinylsiloxane material is allowed for the intraoral application, its protective performance may be questionable. Furthermore, the printing resolution in the z-axis of ACEO technology remains to be 0.4 mm, which causes a visible and perceptible stair-case effect. Thus, the present clinical case highlights a need for an alternative soft biocompatible rubber-like material, which could be printed simultaneously in various hardness grades, in the range from 20A to 90A Shore with a higher resolution in z-axis.

The digital workflow of a MG was reported to be beneficial towards the conventional approach [18,22]. However, until now the evidence to the superior shock absorption of 3D printed MGs is provided only on the experimental in vitro level [23]. Clinical trials are necessary to estimate their protective capacities. Further research on this topic may also consider the wear comfort compared to conventionally produced EVA MG using visual analog scale (VAS), the comparison of manufacturing accuracy using both AM methods and finite element analysis of the MG behaviour during load.

## 4. Conclusions

The pilot clinical case shows an efficient pathway to construct a customized multi-material MG in a digital workflow, starting with the intraoral scanning. It highlights the need for further hardware development. This protocol may become very promising, as soon as another soft biocompatible rubber-like material for simultaneous multi-material printing is introduced to the market.

## Data Availability

The datasets used and/or analysed during the current study are available from the corresponding author on reasonable request. All STL files can be transferred via cloud service on request.
